# Vaccination of contacts of Ebola virus disease survivors to prevent further transmission

**DOI:** 10.1016/S2214-109X(20)30454-X

**Published:** 2020-12

**Authors:** Reena H Doshi, Monica Fleming, Arsene Kabwaya Mukoka, Rosalind J Carter, Terri B Hyde, Mary Choi, Michel Kabamba Nzaji, Stephane Hans Bateyi, Athalia Christie, Stuart T Nichol, Inger K Damon, Michael Beach, Elisabeth Mukamba Musenga, David L Fitter

**Affiliations:** Global Immunization Division, Centers for Disease Control and Prevention, Atlanta, GA 30329, USA; Global Immunization Division, Centers for Disease Control and Prevention, Atlanta, GA 30329, USA; Expanded Programme on Immunization, Kinshasa, Democratic Republic of the Congo; Global Immunization Division, Centers for Disease Control and Prevention, Atlanta, GA 30329, USA; Global Immunization Division, Centers for Disease Control and Prevention, Atlanta, GA 30329, USA; Division of High-Consequence Pathogens and Pathology, National Center for Emerging and Zoonotic Infectious Diseases, Centers for Disease Control and Prevention, Atlanta, GA, USA; Expanded Programme on Immunization, Kinshasa, Democratic Republic of the Congo; Expanded Programme on Immunization, Goma, Democratic Republic of the Congo; Center for Global Health, Centers for Disease Control and Prevention, Atlanta, GA 30329, USA; Division of High-Consequence Pathogens and Pathology, National Center for Emerging and Zoonotic Infectious Diseases, Centers for Disease Control and Prevention, Atlanta, GA, USA; Division of High-Consequence Pathogens and Pathology, National Center for Emerging and Zoonotic Infectious Diseases, Centers for Disease Control and Prevention, Atlanta, GA, USA; Division of Foodborne, Waterborne and Environmental Diseases, National Center for Emerging and Zoonotic Infectious Diseases, Centers for Disease Control and Prevention, Atlanta, GA, USA; Expanded Programme on Immunization, Kinshasa, Democratic Republic of the Congo; Global Immunization Division, Centers for Disease Control and Prevention, Atlanta, GA 30329, USA

On April 10, 2020, just 2 days before the anticipated declaration of the end of the North Kivu and Ituri Ebola virus disease (EVD) outbreak in DR Congo, and 53 days after the last confirmed case of EVD had been reported, a new case was confirmed. Sequencing of patient samples from the case in April and six others that followed indicated that these cases were likely to have come from a reintroduction of the virus from a persistently infected survivor.^1^ This group of cases marked the second flare-up linked to an EVD survivor during this outbreak. In November, 2019, a relapse case in North Kivu resulted in widespread transmission across multiple health zones, helping to extend the outbreak by at least 3 months.

Ebola virus is known to persist in immune-privileged sites in survivors.^2,3^ Viral RNA has been detected in semen up to 2 years after recovery,^2^ and clinical relapse has been documented.^3^ In North Kivu and Ituri, there are 1171 known EVD survivors, and although the vast majority will no longer transmit the virus, transmission events linked to survivors continue to pose a risk.^1^

Unlike previous Ebola outbreaks, the current EVD response strategy has benefited from new investigational tools, including a safe and effective vaccine that protects against Ebola virus.^4^ The rVSVΔG-ZEBOV-GP vaccine has been used since the beginning of the North Kivu and Ituri outbreak under a compassionate use protocol using ring vaccination.^4,5^ The success of ring vaccination is dependent on strong case investigation, contact identification, and follow-up to ensure all contacts are identified. The ring is defined using the list of contact names ascertained and routinely updated by contact tracers during the investigation, and those on the contact list and their contacts (ie, contacts of contacts) are offered the vaccine. Rings are closed when all identified individuals have been offered vaccination or 21 days have passed. Eligible individuals who do not receive the vaccine due to refusal or because they were not listed might not have another opportunity to be vaccinated unless they are part of a newly defined ring. In theory, the majority of contacts and contacts of contacts should have been vaccinated, or at least offered vaccination, while the ring was open. However, during the North Kivu and Ituri outbreak, and now the Equateur outbreak, many new cases have not been previously identified as contacts, suggesting incomplete contact identification. Additionally, the ring vaccination strategy, as currently implemented, does not account for the fact that a survivor’s contacts are dynamic and can change over time.

There is a need to incorporate additional risk reduction strategies, such as regular re-elicitation and vaccination of new and previously unvaccinated contacts of survivors. Offering ongoing vaccination to contacts of survivors early and throughout the outbreak could potentially have prevented both the November, 2019, and April, 2020, flare-ups.

In eastern DR Congo, there is an established survivor programme, which includes dedicated clinics that provide health services, including testing of semen to detect the persistence of Ebola virus, counselling on sexual transmission, and specialised psychological support. Monthly follow-up visits are encouraged for survivors up to 18 months after infection. Survivor clinics present an opportunity to expand vaccination to contacts of survivors. Trained counsellors can work with survivors during monthly clinic visits to reassess close contacts and contacts of contacts. The vaccine should be offered to all newly identified or previously unvaccinated contacts to prevent potential sexual transmission and transmission from a relapse case. In the event of insufficient vaccine supply, a more targeted approach could be considered, such as prioritising vaccination of the contacts of male survivors only, or even just the contacts of Ebola-PCR-positive male survivors. Regardless of the approach, the number of doses needed would be minimal.

Given the stigma around EVD, appropriate and socially sensitive communication strategies need to be developed in consultation with survivor associations to educate survivors and facilitate community engagement. Educating community members and highlighting the protective value of vaccinating contacts and contacts of contacts should reduce the perceived risk of survivors and overall EVD stigma. The elicitation of sexual partners could be challenging because of intimate partner violence, the sexual exploitation of underage girls, and the need to protect partner anonymity. It is important that mitigation policies and procedures designed for HIV partner services be incorporated into survivor clinics. These concerns could be further addressed by eliciting a broader range of contacts without distinguishing between sexual and close contacts and revealing the nature of their contact with the survivor.

In the past 7 years, we have witnessed the two largest and most complex EVD outbreaks in history. Given the continued risk of flare-ups from the North Kivu and Ituri outbreak and, now, the Equateur outbreak, and the likelihood of future EVD outbreaks in DR Congo and elsewhere, response methods need to be updated and improved, and any new tools available need to be strategically used to efficiently stop the prolonged spread of EVD. Transmission events associated with survivors have hampered outbreak containment and highlight the urgent need to incorporate vaccination into survivor programmes as standard practice. It is time to develop, implement, and standardise a protocol for vaccination of contacts of survivors during Ebola outbreaks to prevent survivor-related infections in the future.
